# *Edwardsiella ictaluri* type III secretion system effector EseG modulates cytoskeletal dynamics and immune response in macrophages

**DOI:** 10.1128/iai.00525-24

**Published:** 2025-07-10

**Authors:** Lidiya Dubytska, Ranjan Koirala, Matthew Rogge, Ronald Thune

**Affiliations:** 1 Department of Biological Sciences and Chemistry, Southern University and A&M College 14694 , Baton Rouge, Louisiana, USA; 2 Department of Biology, University of Wisconsin-Stevens Point 14756, Stevens Point, Wisconsin, USA; 3 Department of Pathobiological Sciences, Louisiana State University School of Veterinary Medicine 70164, Baton Rouge, Louisiana, USA; University of Pennsylvania School of Veterinary Medicine, Philadelphia, Pennsylvania, USA

**Keywords:** *Edwardsiella ictaluri*, macrophage, type III secretion system, effector EseG, cytoskeletal dynamics, immune response

## Abstract

*Edwardsiella ictaluri* is a gram-negative enteric pathogen responsible for enteric septicemia of catfish. One of the critical virulence factors identified in *E. ictaluri* is its type III secretion system (T3SS). In this study, we report that the T3SS effector protein EseG requires the small chaperone protein EscB for translocation. EseG shows partial homology to two *Salmonella* T3SS effectors, SseG and SseF, as well as to the *Edwardsiella piscicida* effector EseG, all of which also require chaperones for translocation. Functionally, EseG interacts with and inactivates Ras homolog family member A (RhoA), a small GTPase that regulates the dynamic organization of the microtubule and actin cytoskeleton. The cytoskeleton is vital for cell morphology, polarity, adhesion, exocytosis, endocytosis, cytokinesis, and transcriptional control. We demonstrate that inactivation of RhoA by EseG leads to the disassembly of both the microtubule and actin cytoskeleton. Moreover, EseG was found to modulate immune responses by altering the expression of several pro-inflammatory interleukins and transcription factors, as well as cyclooxygenase-2 (COX-2). Reduced expression of COX-2 leads to decreased production of prostaglandin E2, a key mediator of inflammation. Additionally, a Δ*eseG* mutant strain exhibited reduced virulence and persistence in channel catfish (*Ictalurus punctatus*), underscoring the importance of EseG in the disease process. Collectively, our data highlight EseG as a critical factor in *E. ictaluri* pathogenesis, particularly in its ability to modulate host immune responses. By elucidating the function of EseG, this study contributes to a deeper understanding of *E. ictaluri* pathogenesis.

## INTRODUCTION

*Edwardsiella ictaluri* is a gram-negative intracellular pathogen responsible for enteric septicemia in commercially important channel catfish (*Ictalurus punctatus*) (1). In many gram-negative pathogens, type III secretion systems (T3SS) are an important component of virulence (2, 3). Previous studies have shown that a T3SS is essential for *E. ictaluri* virulence and intracellular replication, and that it plays a significant role in inducing programmed cell death in host cells (4–6). The T3SS functions by delivering effector proteins directly into infected eukaryotic cells (2, 7), and these effector proteins often mimic eukaryotic proteins in structure and function, targeting a variety of host physiological processes (8).

The concerted action of these effectors stimulates or interferes with host cellular processes, thereby dictating the terms of bacterium-host cell interactions (3). In *Salmonella* spp., more than 30 effectors are secreted by two different T3SSs. These effectors are involved in several diverse functions, such as forced entry into epithelial cells, intracellular replication inside vacuoles, and suppression of cellular immune responses (9–11). In *Yersinia* species, six Yop effectors were identified and found to disrupt vital signaling cascades that are required for innate immunity (12, 13). T3SS effectors are generally less conserved than other components of T3SSs, and they perform unique functions that define a given pathogen’s virulence strategy.

We have previously identified nine T3SS effectors in *E. ictaluri* and demonstrated their translocation into host cells and their role in intracellular replication (7). Notably, one of these effectors, EseN, functions as a phosphothreonine lyase involved in modulating immune responses and signaling pathways (14–16). The function of the other eight effectors remains unknown.

The *E. ictaluri* T3SS effector EseG is similar to *Salmonella* effectors SseF and SseG, which are translocated into host cells by the *Salmonella* T3SS2 (17–19). These effectors localize to the membranes of *Salmonella*-containing vacuoles (SCVs) and are involved in the formation of *Salmonella*-induced filaments (SIFs), crucial for SCV maturation (19–21). Additionally, *E. ictaluri* EseG is homologous to the *Edwardsiella piscicida* (formerly classified as *Edwardsiella tarda* [22–27]) T3SS effector EseG. In *E. piscicida*, EseG localizes to the *E. piscicida*-containing vacuoles after translocation and disassembles microtubules when overexpressed in mammalian cells (28, 29), which contributes to the virulence of *E. piscicida* in fish.

Rho GTPases play a central role in the regulation of host cytoskeleton dynamics and in numerous signal transduction pathways and are common targets for bacterial effectors (30–39). These small GTPases are critical for membrane trafficking, vesicle biogenesis, cargo transport, and fusion events (30, 40–42). Rho GTPases, such as Cdc42, Rho, and Rac, act as molecular switches, cycling between inactive GDP-bound and active GTP-bound states. When active, they interact with over 30 eukaryotic effector proteins to initiate downstream signaling (38, 43). Bacterial effectors like *Salmonella* SopE and SopE2 function as guanine-nucleotide exchange factors (GEFs) to catalyze GDP-to-GTP exchange in Cdc42 and Rac1 (44, 45), despite having no sequence or structural homology to eukaryotic GEFs.

In *E. ictaluri*, EseG is located downstream of EscB, a small chaperone-like protein. Our study demonstrates that EscB is required for the translocation of EseG, likely serving as its chaperone, similar to the chaperones for SseF and SseG in *Salmonella* and EseG of *E. piscicida* (28, 46). Functional studies in our lab revealed that *E. ictaluri* EseG interacts with and inactivates RhoA, a Rho GTPase responsible for organizing the actin cytoskeleton, including polymerization, stress fiber formation, microtubule dynamics, and cellular processes like adhesion, motility, cytokinesis, and transcriptional control.

We have further shown that EseG disassembles both the microtubule and actin cytoskeleton, likely as a result of RhoA inactivation. This disruption of cytoskeletal organization, combined with possible alterations in signal transduction, leads to immune modulation, as evidenced by changes in pro-inflammatory interleukins (ILs) and transcription factor expression. Additionally, a Δ*eseG* mutant displayed reduced virulence and persistence in channel catfish, highlighting the importance of EseG in the pathogenesis of *E. ictaluri*.

## RESULTS

### The EscB is required for EseG translocation

**Fig 1:**
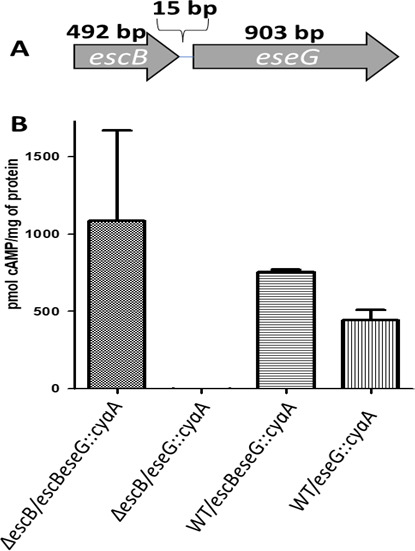
(A) EseG and its chaperone EscB are translated separately. The *escB* and *eseG* genes are separated by only 15 bp from the first start codon ATG. (B) EseB is a required chaperone for translocation of EseG. Localization of the *escB* gene is not important for EseG translocation, but is essential for the efficiency of effector translocation. *N* = 3.

The *escB* gene is upstream of *eseG* and is transcribed in the same direction in the *E. ictaluri* genome, with a 15 base pair (bp) gap between the stop codon of *escB* and the start codon of *eseG* (Fig. 1A). EscB is a small 18.2 kDa polypeptide with a isoelectric point (pI) of 4.58, characteristics common to most chaperones (47). Furthermore, it has a tetratricopeptide repeat domain (FTFAFACVLQQQQEYRQALTLFSYSLALQAND), as identified by a Pfam scan (http://pfam.janelia.org/). This domain is also present in the LcrH/SycD-like chaperones (48, 49).

In the cyclic AMP (cAMP) production in the translocation assay (Fig. 1B), head kidney-derived macrophage (HKDM) cells infected with the wild type (WT) strain harboring either *escBeseG::cyaA* or *eseG::cyaA*, the EseG::cyaA fusions were successfully translocated into the cytoplasm of infected HKDM cells. However, when *escB* was mutated, translocation of the EseG::cyaA fusion occurred only from the *escBeseG::cyaA* construct. This indicates that EscB is required for EseG translocation and that the plasmid-encoded *escB* can complement the chromosomal deletion of *escB* in the mutant.

### Sequence analysis of EseG

EseG is a 300-amino-acid protein encoded within the *E. ictaluri* T3SS pathogenicity island (7). Based on computational analysis (https://web.expasy.org/cgi-bin/protparam/protparam), EseG is classified as stable and shows homology to hypothetical proteins from *Aeromonas diversa* (94% coverage, 50% identity, 63% similarity), *Escherichia coli* (63% coverage, 38% identity, 55% similarity), and *Yersinia enterocolitica* (68% coverage, 26% identity, 46% similarity). Additionally, EseG shares significant homology with the *Edwardsiella piscicida* T3SS EseG protein (100% coverage, 71% identity, 79% similarity) and two *Salmonella* T3SS effector proteins: SseG (33% coverage, 39% identity, 57% similarity) and SseF (54% coverage, 32% identity, 47% similarity), which are critical for pathogen-containing vacuole (PCV) maturation during infection. This suggests that *E. ictaluri* T3SS effector EseG may play a similar role in its virulence.

**Fig 2:**
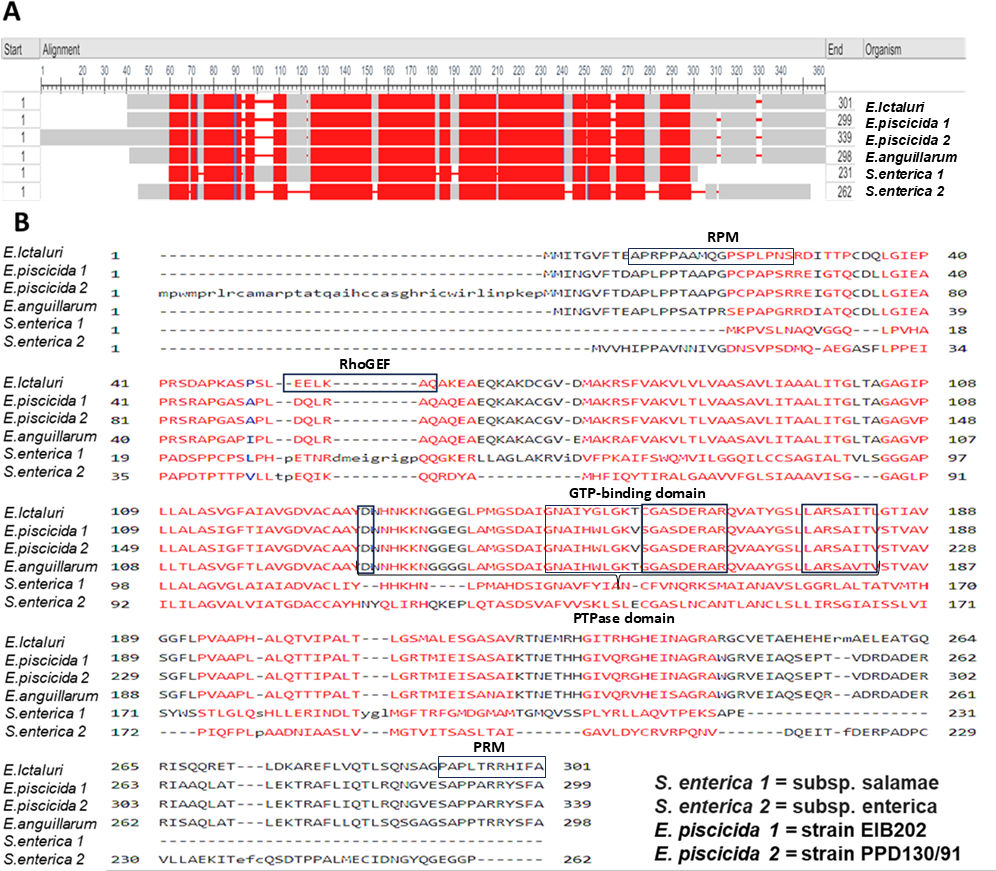
(A) Conserved regions of EseG homologs. (B) *Edwardsiella ictaluri* EseG sequence alignment with different EseG homologs. Predicted conserved regions are highlighted in red, predicted conserved domains are boxed. The Lx₂Rx₄L motif directs the effector protein to perinuclear vesicles, D(X)28HC(X)5R--a protein tyrosine phosphatase (PTPase) domain, EELKAQ--RhoGEF domain, GNAIYGLGKTCGASDER- -GTP-binding domain, "PRPPAAMQGPSPLPNS" and "PAPLTRRHSFA"--proline-rich motif (PRM).These unique domains suggest a specialized function for EseG in *E. ictaluri* virulence.

Interestingly, EseG and its homologs share conserved regions (Fig. 2A, red boxes). In *Salmonella*, this conserved region is used by SseF to recruit dynein to the SCV and by SseG to target the Golgi complex, facilitating *Salmonella*’s vigorous intracellular multiplication (50). However, *Salmonella* SseG’s Golgi-targeting region shares only 89% coverage and 51% identity with *E. ictaluri* EseG, and *E. piscicida* EseG’s actin-binding domain shares only 50% coverage and 52% identity with that of *E. ictaluri*.

Despite these differences, all the described *E. ictaluri* EseG homologs are involved in PCV maturation and cytoskeletal reorganization. Moreover, EseG contains several important domains (Fig. 2B), including the Lx2Rx4L domain, which can target effectors to a perinuclear vesicle (8), and resembles the 87–143 aa region of *Salmonella* SseG responsible for Golgi targeting (8, 51). EseG also has a D(X)28HC(X)5R protein tyrosine phosphatase (PTPase) domain (8) present in all *Edwardsiella* species but absent in *Salmonella*, and an EELKAQ RhoGEF domain (8) found only in *E. ictaluri*.

The motif “GNAIYGLGKTCGASDER” present in the EseG sequence resembles part of a GTP-binding domain commonly found in GTPases and hydrolyzes GTP, playing key roles in signal transduction, cell division, and intracellular transport. The presence of the “GLGK” segment in this motif suggests it could bind nucleotides like GTP (52, 53). Proteins with GTP-binding motifs, like GTPases, often interact with GTPase-activating proteins (GAPs) and GEFs (52, 53). Segments such as “PRPPAAMQGPSPLPNS” and “PAPLTRRHSFA” contain proline-rich motifs that are known to mediate protein-protein interactions, often serving as binding sites for proteins with SH3 or WW domains. These domains are found in signaling proteins or cytoskeletal components (54, 55), and may interact with proteins like vinculin, talin, and profilin that modulate actin filaments and cellular structure (56, 57).

### EseG inactivates RhoA

The Swiss-Model (58) predicts that one of EseG’s potential functions is as a Rho-GEF. Sequence analyses of EseG suggest that EseG can function as a GTPase that hydrolyzes GTP and GAPs. Alternatively, GEFs could be potential target proteins. Because GEFs activate Rho GTPases and the Rho GTPase RhoA is crucial for regulating various cellular processes, including actin/microtubule reorganization (32, 33, 38, 59–63), we investigated whether EseG is involved in RhoA activation.

**Fig 3:**
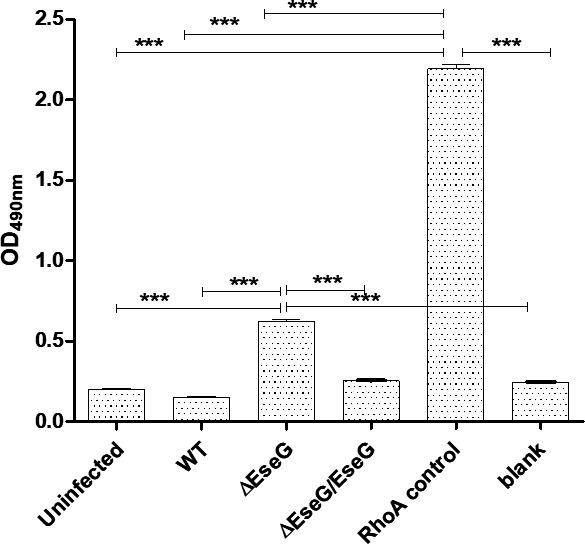
EseG inhibits RhoA in infected HKDM. Representative experiment of three independent experiments. To reduce RhoA activation, HKDMs were seeded in serum-free medium and infected with *E. ictaluri* WT (WT), EseG mutant (*∆eseG*), or EseG mutant complement (*∆eseG/eseG*) for 5 h. To detect RhoA activity, the RhoA G-LISA kit was used. Data are presented as means ± SD. Statistical analyses were performed using one-way analysis of variance with the Bonferroni post-test. * = *P* ≤ 0.05; ** = *P* ≤ 0.01; *** = *P* ≤ 0.01. *P*-values were obtained using the *t*-test method. *N* = 3.

Using the RhoA G-LISA kit, RhoA activity was measured in uninfected HKDM and HKDM infected with *E. ictaluri* WT and *eseG* mutant strains. Both uninfected HKDM and HKDM infected with *E. ictaluri* WT displayed baseline RhoA activity (Fig. 3). However, RhoA activity in HKDM infected with the *eseG* mutant was significantly elevated compared to uninfected and WT-infected cells, as well as the blank control. EseG complementation restores WT phenotype. This suggests that *E. ictaluri* infection activates RhoA, and that EseG, unlike typical GEFs, functions to inactivate RhoA, indicating a role in regulating RhoA activity during infection.

### EseG interacts with RhoA

**Fig 4:**
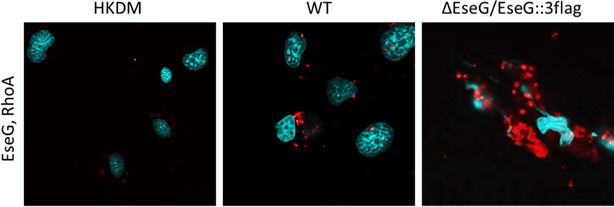
Interaction between RhoA and *E. ictaluri* type III secretion system effector EseG in HKDMs using the PLA with mouse monoclonal antibody to EseG::3Flag and rabbit polyclonal antibody to RhoA as the primary antibodies. Interactions were detected using anti-mouse and anti-rabbit PLA probes as the secondary antibodies. Positive interactions of the PLA probes are represented by red fluorescent dots and are present in HKDMs infected with EseG::3Flag *E. ictaluri*, on background level in the *E. ictaluri* WT and uninfected HKDM strains. Nuclei stained with 4′,6-diamidino-2-phenylindole (DAPI) (blue). *N* = 3

The proximity ligation assay (PLA) results for RhoA and EseG are shown in Fig. 4. A background of red fluorescent dots is present in HKDMs infected with the WT strain. However, in HKDMs infected with the ∆*eseG* mutant harboring the *eseG::3flag* construct, an additional strong red fluorescent signal indicates a positive interaction between EseG and RhoA. This suggests a specific interaction between EseG and RhoA.

### EseG is involved in α/β-tubulin destabilization

**Fig 5:**
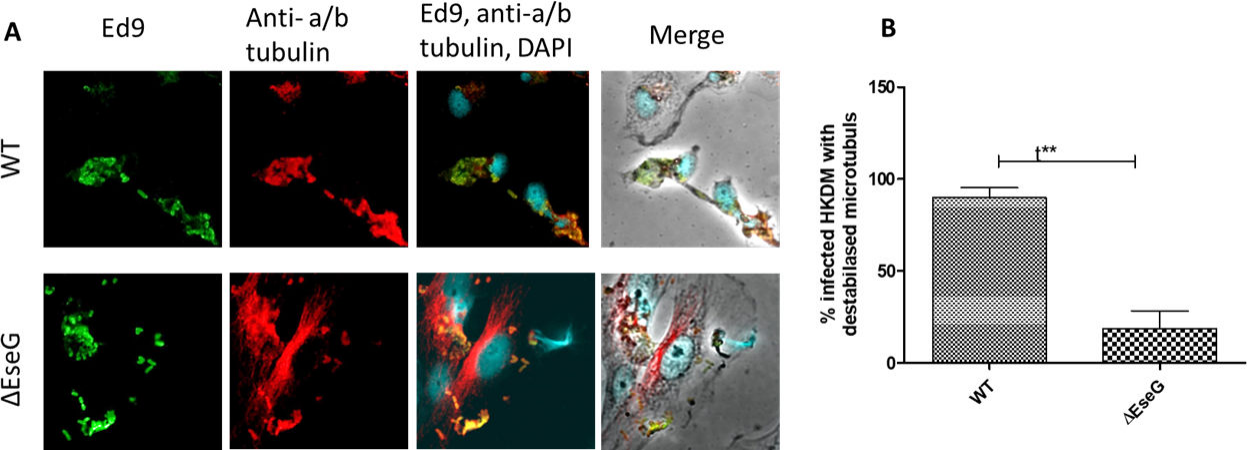
EseG is involved in microtubule destabilization. (**A**) Immunofluorescence visualization of microtubule destabilization by EseG. *Edwardsiella ictaluri* are labeled with an anti-*E. ictaluri* Ed9 antibody (green) and an anti-α/β-tubulin antibody (red), respectively. The nucleus is labeled with 4′,6-diamidino-2-phenylindole (DAPI) (blue). (**B**) Measurement of microtubule destabilization in HKDM cells infected with *E. ictaluri* WT and ∆*eseG* mutant. One hundred twenty infected cells were counted in at least three independent experiments. Data are presented as means ± SD. **P ≤ 0.01. *P*-values were obtained using the *t*-test method. *N* = 3.

To assess the role of EseG in α/β-tubulin destabilization, HKDM cells were infected with either *E. ictaluri* WT or Δ*eseG* strains for 5 h and subsequently stained with anti*-E*. *ictaluri* (green) and anti-α-tubulin (red) antibodies. Results shown in Fig. 5A indicate that HKDM infected with *E. ictaluri* WT exhibited significant microtubule destabilization, while cells infected with Δ*eseG* maintained normal filamentous microtubules. As presented in Fig. 5B, nearly 90% of cells infected with *E. ictaluri* WT displayed microtubule destabilization, whereas the deletion of *eseG* reduced this percentage to less than 20%.

**Fig 6:**
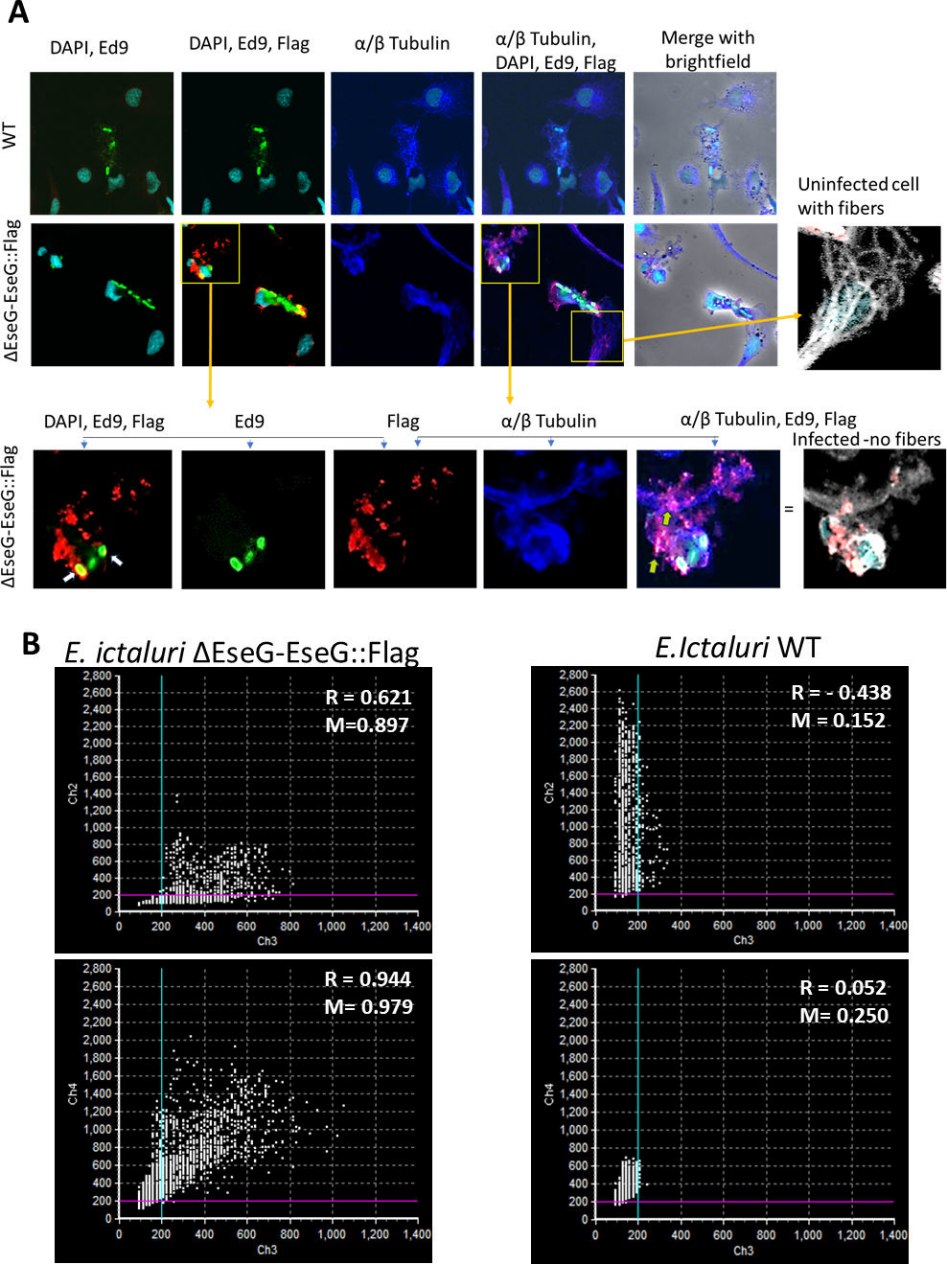
EseG colocalizes with microtubules and the Edwardsiella-containing vacuole (ECV). (**A**) *Edwardsiella ictaluri* are labeled with the anti-*E*. *ictaluri* Ed9 monoclonal antibody (green), microtubules are labeled with an anti-α/β-tubulin antibody (blue), and Δ*eseG*::3XFlag is labeled with anti-Flag (red). Nucleus labeled with 4′,6-diamidino-2-phenylindole (DAPI) (light blue). Yellow indicates colocalization of *E. ictaluri* and EseG indicated by white arrows. Pink indicates colocalization of EseG and tubulin (green arrows). Gray pictures demonstrate microtubule destabilization in HKDM infected with ∆*eseG* complemented with *eseG::3flag*, and microtubule fibers in uninfected cells from the same slide. *N* = 3. (**B**) Scatterplot of green (channel 2, *E. ictaluri*), red (channel 3, EseG::Flag), and blue (channel 4, α/β-tubulin). Numbers represent Pearson’s correlation coefficient (*R*) and Manders’ overlap coefficient (*M*). WT acts as a negative control which lacks EseG::Flag signal. FLAG-expressing samples showed increased colocalization compared to WT negative controls, supporting the presence of spatial association of the FLAG-tagged protein with the signals under study.

To determine the colocalization of EseG with α/β-tubulin and its intracellular localization, HKDM cells were infected with WT and Δ*eseG* strains expressing EseG::3Flag for 5 h. Cells were then stained with anti-flag (red), anti-α/β-tubulin (cyan), and anti-*E*. *ictaluri* (green) antibodies. As illustrated in Fig. 6, HKDM infected with *E. ictaluri* WT demonstrated microtubule destabilization and did not express EseG::3Flag. HKDM cells infected with ∆*eseG* complemented with EseG::3Flag also demonstrate microtubule destabilization, indicating a specific role of EseG in this process (Fig. 6 gray pictures). To investigate whether FLAG-tagged EseG in infected HKDM localizes to the α/β-tubulin, we performed colocalization analysis. Regions imaged from FLAG-EseG-expressing cells showed markedly higher Pearson’s coefficient (*R* = 0.944), indicating a strong correlation in signal intensity between FLAG and α/β-tubulin. Manders’ overlap coefficient also increased significantly (*M* = 0.979), suggesting that a large proportion of the FLAG-EseG signal physically overlapped with the α/β-tubulin. In contrast, *R* = 0.05 was observed in HKDM infected with the WT negative control that does not express Flag, indicating no spatial correlation between the signals in these channels. Manders’ coefficients in these controls were similarly low (0.250), supporting minimal or no signal overlap (Fig. 6B). These data support the conclusion that FLAG-EseG localizes to α/β-tubulin, while the negative controls confirm the specificity of the colocalization.

In addition, colocalization analysis of EseG with *E. ictaluri* located in ECV in EseG::Flag-infected HKDM cells also showed spatial association, with *R* = 0.621 and *M* = 0.897, indicating strong colocalization between the tagged protein and *E. ictaluri*. HKDMs infected with WT that serve as a negative control demonstrate lower Pearson’s coefficient (*R* = −0.438), indicating no spatial correlation between the signals in these channels. Manders’ coefficients in these controls were similarly low (0.152), supporting no signal overlap (Fig. 6B). This suggests that FLAG-tagged EseG shows spatial association with the ECV, which is absent in the wild-type background.

### EseG is involved in actin reorganization

**Fig 7:**
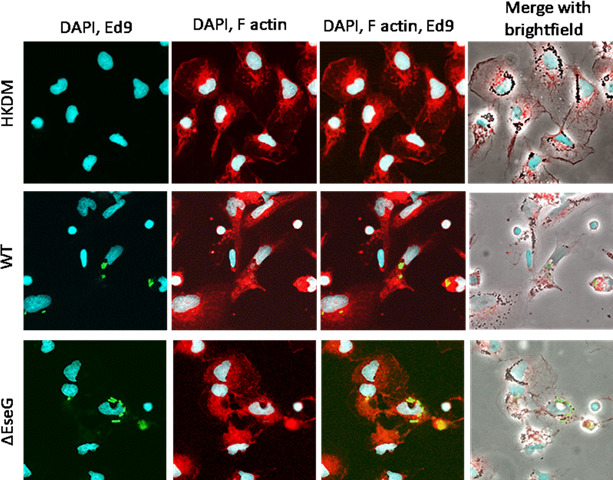
EseG is involved in actin reorganization. *Edwardsiella ictaluri* is labeled with the monoclonal anti-*E*. *ictaluri* Ed9 antibody (green). Actin is stained with phalloidin (red). Nuclei are labeled with 4′,6-diamidino-2-phenylindole (DAPI) (blue). Yellow color indicates colocalization of *E. ictaluri* and F-actin. *N* = 3.

**Fig 8:**
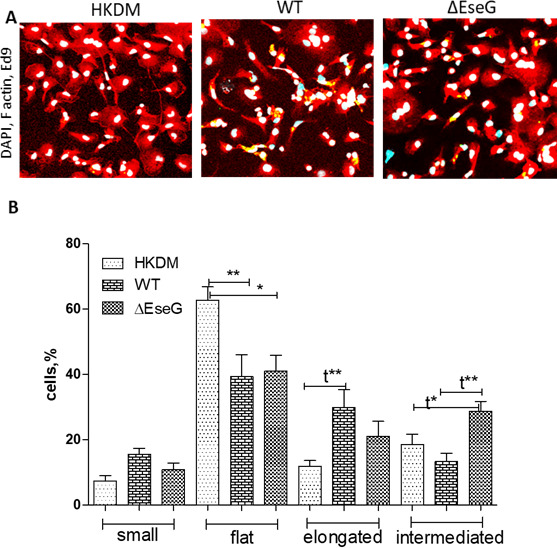
(**A**) EseG is involved in actin reorganization. *Edwardsiella ictaluri* is labeled with an anti-*E. ictaluri* Ed9 antibody (green). Actin is stained with phalloidin (red). The nucleus is labeled with 4′,6-diamidino-2-phenylindole (DAPI) (blue). Yellow color indicates colocalization of *E. ictaluri* and F-actin. (**B**) Percentage of cells with different morphology in HKDM infected with *E. ictaluri* WT and Δ*eseG*. More than one hundred cells were counted for each treatment in at least three independent experiments. Data are presented as means ± SD. Comparison between groups was based on one-way analysis of variance with the Bonferroni procedure for comparison of group means. Comparison between two groups was also analyzed by *t*-test (t); *P < 0.05, **P < 0.01, ***P < 0001. *N* = 3.

Given that small GTPase RhoA and its downstream effectors are key regulators of the actin cytoskeleton and related functions in eukaryotic cells, we investigated the role of EseG in HKDM actin reorganization. As shown in Fig. 7, HKDM cells infected with *E. ictaluri* WT exhibited destabilization of actin structures compared to uninfected HKDM cells. In contrast, HKDM cells infected with the Δ*eseG* strain displayed actin structures distinct from both uninfected HKDMs and those infected with the WT strain. Notably, the shape of HKDM cells was affected whenever WT or Δ*eseG* strains were examined (Fig. 8A and B).

Our analysis revealed that the percentage of flat cells was significantly higher in uninfected HKDMs compared to those infected with either the WT or Δ*eseG* strains (Fig. 8B). The presence of elongated cells was significantly higher in HKDM infected with the WT strain compared to uninfected HKDMs, but there was no significant difference between HKDMs infected with the WT and Δ*eseG* strains. Conversely, the proportion of intermediate-shaped cells was significantly higher in HKDM infected with Δ*ese*G compared to both uninfected HKDMs and those infected with the WT strain. These data suggest that EseG plays a crucial role in actin/cytoskeleton organization.

### *E. ictaluri* effector EseG modulates SP2 and ATF3 transcription factor expression in infected HKDM

**Fig 9:**
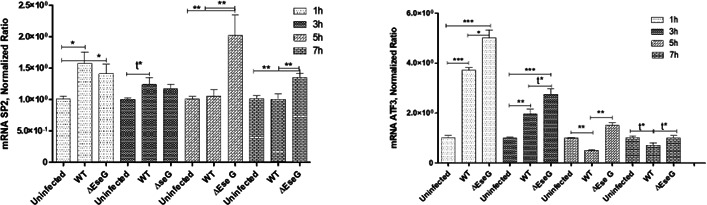
Transcription factor expression detected by reverse transcriptase-qPCR. Data were analyzed by Roche LightCycler 96 qPCR software using the relative expression method. CanX was used as a reference gene. Uninfected, uninfected HKDM; WT, HKDM infected with WT; *∆*EseG, HKDM infected with *∆eseG*. Comparison between groups was based on one-way analysis of variance with the Bonferroni procedure for comparison of group means. Each column represents the mean ± SE of three or four independent experiments. Comparison between two groups was also analyzed by *t*-test (t); *P < 0.05, **P < 0.01, ***P < 0001.

As shown in Fig. 9, SP2 was significantly downregulated in HKDM infected with the WT strain compared to HKDM infected with the Δ*eseG* strain at both 5 and 7 h post-infection (PI). Similarly, ATF3 exhibited significant downregulation in HKDM infected with the WT strain compared to HKDM-∆*eseG* across all time points examined.

Interestingly, both SP2 and ATF3 were upregulated in HKDM infected with the WT strain compared to uninfected HKDMs at 1 and 3 h PI. However, by later time points, SP2 levels returned to an uninfected level, while ATF3 was significantly lower than in uninfected HKDMs. These findings indicate that EseG plays a role in modulating the expression of these transcription factors during infection.

### Effect of *E. ictaluri* T3SS effector *EseG* on modulation of pro- and anti-inflammatory cytokine expression in HKDM

**Fig 10:**
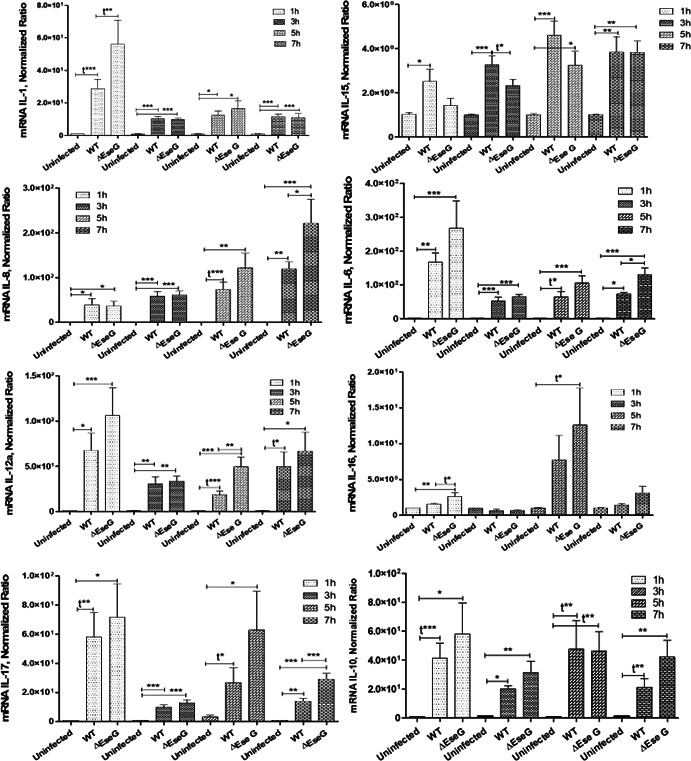
Cytokine expression detected by RT-qPCR. Data were analyzed by Roche LightCycler 96 qPCR software using the relative expression method. CanX was used as a reference gene. Uninfected, uninfected HKDM; WT, HKDM infected with WT; ∆EseG, HKDM infected with ∆*eseG*. Comparison between groups was based on one-way analysis of variance with the Bonferroni procedure for comparison of group means. Each column represents the mean ± SE of four or five independent experiments. Comparison between two groups was also analyzed by *t*-test (t). *P < 0.05, **P < 0.01, ***P < 0001.

RhoA is recognized as a key regulator of the transcription factor Nuclear Factor kappa B (NF-κB) and cytokine expression (64) (Fig. 10). Thus, we assessed the expression of pro-inflammatory (IL-1, IL-6, IL-8, IL-12a, IL-15, IL-16, IL-17) and anti-inflammatory (IL-10) cytokines via reverse transcriptase (RT)-qPCR in HKDM with WT and ∆*eseG* strains at various time points PI, using uninfected HKDM as a negative control.

*E. ictaluri* WT significantly upregulated IL-1 mRNA expression in infected HKDM throughout all examined PI times. Infection with the ∆*eseG* mutant resulted in significantly greater expression of IL-1 compared to WT infection, only at 1 h PI, after which IL-1 levels declined to those seen in HKDMs infected with WT.

Production of IL-15 mRNA in HKDM infected with the WT significantly increased at 1, 3, 5, and 7 h PI compared to uninfected HKDM, while IL-15 levels in ∆*eseG-*infected HKDM significantly decreased compared to WT-infected HKDM at 3 h PI.

*E. ictaluri* WT also stimulated IL-8 production in HKDM at all PI time points. However, cells infected with the ∆*eseG* mutant exhibited significantly higher IL-8 levels than those infected with WT only at 7 h PI. No significant differences in IL-8 production were observed between WT- and Δ*eseG-*infected cells at 1, 3, or 5 h PI.

HKDM infection with WT bacteria induced IL-6 production at all time points. In contrast, cells infected with the ∆*eseG* exhibited higher IL-6 production compared to cells infected with the WT at 1, 5, and 7 h PI, with the difference being significant only at 7 h PI.

Our data indicated a significant upregulation of IL-12a in HKDM infected with the WT compared to uninfected HKDM at all time points. A significant increase in IL-12a was also observed in ∆*eseG-*infected compared to WT-infected cells, but only at 5 h PI.

Production of IL-16 in HKDM infected with the ∆*eseG* significantly increased at 1 h PI compared to uninfected HKDM and HKDM infected with the WT. Levels of IL-16 were also higher in HKDM infected with the ∆*eseG* and HKDM infected with the WT at 5 h PI, with the difference being significant only for ∆eseG-infected HKDM.

IL-17 production in HKDM infected with the WT was significantly higher than in uninfected HKDM during all time points, and in ∆*eseG-*infected HKDM, IL-17 levels were significantly elevated compared to WT-infected cells at 7 h PI.

No significant differences were observed in anti-inflammatory IL-10 expression between WT- and ∆*eseG*-infected HKDM at any time point. However, IL-10 levels were elevated in HKDM infected with the WT compared to uninfected HKDM.

### EseG modulates cyclooxygenase-2 (COX-2) and prostaglandin expression in HKDM

The inducible form of cyclooxygenase is COX-2, which forms prostaglandins from arachidonic acid. In response to inflammatory and other physiologic stimuli and growth factors, the expression of COX-2 is upregulated and is involved in the production of those prostaglandins that mediate pain and support the inflammatory process. Therefore, we first studied COX-2 mRNA expression in HKDM infected with the WT compared to HKDM infected with the ∆*eseG*. We used uninfected HKDM as a negative control.

**Fig 11:**
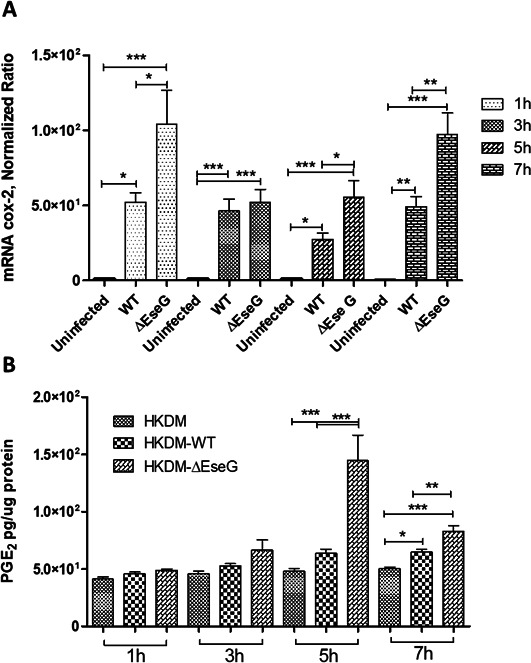
Expression of COX-2 mRNA and PGE2. (**A**) Data were analyzed by Roche LightCycler 96 qPCR software using relative expression method. CanX was used as a reference gene. Uninfected, uninfected HKDM; WT, HKDM infected with WT; *∆*eseG, HKDM infected with *∆eseG*. Each column represents the mean ± SE of three or four independent experiments (depending on time points of infection). Comparison between groups was based on one-way analysis of variance with the Bonferroni procedure. Each column represents the mean ± SE; *P < 0.05, **P < 0.01, ***P < 0001. (**B**) Representative experiment from three independent experiments. Data represent levels of PGE2 expression in uninfected HKDM (uninfected): HKDM infected with WT (WT); HKDM infected with *∆eseG* (*∆eseG*). Comparison between groups was based on one-way analysis of variance with the Bonferroni procedure. Each column represents the mean ± SE; *P < 0.05, **P < 0.01, ***P < 0001.

As illustrated in Fig. 11A, COX-2 mRNA expression was upregulated in WT-infected compared to uninfected HKDM during all times PI and in ∆*eseG-*infected compared to WT-infected HKDM at 1 h, 5 h, and 7 h PI. Furthermore, the analysis of the ability of COX-2 activity to produce prostaglandin E2 (PGE2) (Fig. 11B) revealed that the PGE2 levels significantly increased in HKDM infected with the ∆*eseG* at 5 h and 7 h PI compared to WT-infected HKDM. Similarly, for COX-2 mRNA expression, levels of PGE2 in ∆*eseG*-infected cells were significantly higher than in WT-infected cells.

### EseG plays an important role in virulence

**Fig 12:**
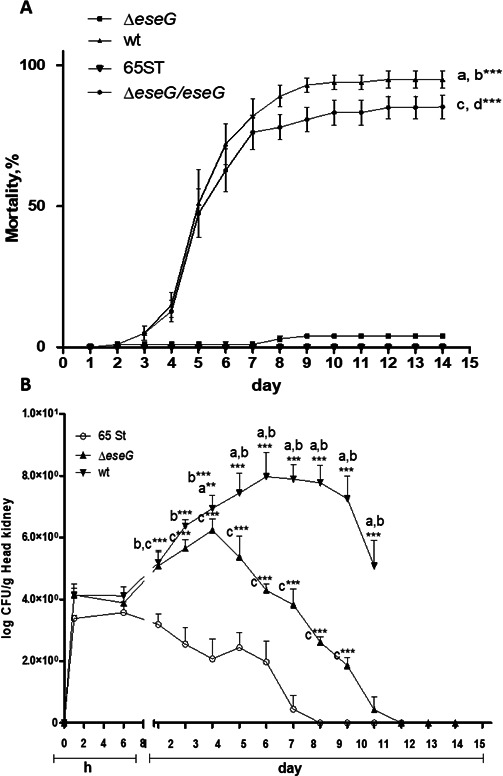
(A) Role of EseG in virulence. Mortality of channel catfish following a 1 h immersion challenge with wild-type *E. ictaluri* (WT)*, eseG mutant* (Δ*eseG), eseG* mutant complement (∆*eseG/eseG*), and a T3SS knockout mutant, 65ST. Each treatment was replicated in four separate tanks, with 25 fish per tank. Differences between treatments were analyzed using one-way ANOVA, followed by Tukey’s *post-hoc* test for pairwise comparisons. Mean ± SD (***P < 0.001). An “a” indicates significant differences between WT and Δ*eseG* mutant, “b” indicates significant differences between WT and 65ST, “c” indicates significant differences between ∆*eseG/eseG* and Δ*eseG*, and “d” indicates significant differences between ∆*eseG/eseG* and 65ST. *N* = 3. (B) Persistence of *E. ictaluri* strains in the channel catfish head kidney following a 30 min immersion challenge with either WT, Δ*eseG,* or 65ST strains. Data are reported as colony-forming units (CFU) per gram of head kidney and are presented as the mean ± SD of quadruplicate samples per group. Data were analyzed by two-way ANOVA followed by Bonferroni’s *post hoc* test to determine significance of the data for the mutant compared to the WT (*P < 0.05, **P < 0.01, ***P < 0.001). An “a” indicates significant differences between WT and Δ*eseG* mutant, “b” indicates significant differences between WT and 65ST, and “c” indicates significant differences between Δ*eseG* and 65ST. *N* = 3.

As indicated in Fig. 12A, Δ*eseG* is significantly attenuated compared to the WT strain. The T3SS knockout mutant 65ST, which was previously shown to be completely avirulent (5), again demonstrated that same phenotype. Importantly, complementation of the ∆*eseG* mutant restores the WT phenotype, indicating the specific role of EseG in this process.

As seen in Fig. 12B, following immersion challenge, no significant difference was found between the numbers of colony-forming units per gram (CFU/g) tissue for 8 h. Numbers of the 65ST T3SS mutant were significantly lower than WT and Δ*eseG* on days 1 through day 9 for the *eseG* mutant and day 10 for WT. The Δ*eseG* strain was not significantly lower than WT up to day 3, but was reduced through the remainder of the experiment, suggesting a role for other T3SS effectors in persistence within the head kidney.

## DISCUSSION

*Edwardsiella ictaluri,* a member of the *Hafniaceae* family, is closely related to other enteric pathogens, such as other *Edwardsiella* species, *Escherichia coli* strains, and pathogenic *Yersinia* and *Salmonella* species. These pathogens are associated with various human diseases, such as typhoid fever, diarrheal illnesses, and bubonic plague (3, 28). *Edwardsiella ictaluri* causes enteric septicemia in commercially valuable catfish, *Ictalurus punctatus*, resulting in significant economic losses for the aquaculture industry (1).

A key virulence factor shared among these pathogens is the T3SS. *Edwardsiella ictaluri* survives and replicates in channel catfish HKDMs (6, 65, 66). This ability to persist within HKDM is attributed to a variety of virulence factors, including an acid-activated urease (65) and a *Salmonella* pathogenicity island 2-like T3SS (T3SS2) (67). Further progression of infection is driven by bacterial effector proteins that are translocated into the host cell cytoplasm via the T3SS (7). These translocated proteins interact with specific host cell targets, disrupting host defense mechanisms and manipulating host cell processes to the pathogen’s advantage (67, 68).

*Edwardsiella ictaluri* EseG is similar to *Salmonella* SseF and SseG (9), which are secreted by the T3SS2 *in vitro* and translocated into the host cell *ex vivo* (17, 18). Both effectors are associated with membranes of SCVs and are necessary for the formation of SIFs that are important for SCV maturation (19–21). SseF and SseG colocalize with microtubules in infected HeLa cells, and this complex serves as a scaffold for SIF formation (69). In addition, SseG and SseF physically interact with each other and are important for targeting the SCV to the Golgi network in infected epithelial cells (17, 19, 21, 69, 70), where an intact Golgi network is required for intracellular replication of *Salmonella* (51).

A predicted transmembrane domain is located in the middle of SseG and is responsible for targeting SseG specifically to the trans-Golgi network. *Salmonella* replication and maturation of the SCV also require selective interactions with the endocytic and secretory pathways (70). *Edwardsiella ictaluri* EseG also contains three transmembrane domains but lacks the domain targeting the trans-Golgi network that is present in *Salmonella* SseG. This indicates that EseG has membrane localization, similar to SseF and SseG, but has a different mechanism of action.

*E. ictaluri* EseG is also similar to *E. piscicida* T3SS effector EseG. *E. piscicida* is a gram-negative enteric pathogen that also causes hemorrhagic septicemia in fish. *E. piscicida* EseG is localized to the *E*. piscicida-containing vacuoles and is able to disassemble microtubule structures when overexpressed in mammalian cells (28, 29). *Edwardsiella ictaluri* EseG homologs SseG, SseF, and EseG require a chaperone for translocation (28, 46). We have shown that *E. ictaluri* EscB has characteristics common to most chaperones and is required for EseG translocation (Fig. 1).

The *Salmonella* SseG Golgi-targeting region shares only 89% of coverage and 51% identity with the *E. ictaluri EseG*. Similarly, the *E*. piscicida EseG actin-binding domain demonstrates only 50% coverage with 52% identity. Interestingly, *E. ictaluri* EseG contains an Lx2Rx4L domain that targets effectors to a perinuclear vesicle (8), a D(X)28HC(X)5R catalytic PTPase domain present in YopH in *Yersinia* (8), and the catalytic domain EELKAQ found in the Rho-GEF domain (8) (Fig. 2). Based on these domains, EseG potential target proteins include GAPs, GEFs, SH3 or WW domain-containing proteins, membrane receptors (like G protein-coupled receptors [GPCRs] or integrins), actin-binding proteins, like vinculin, profilin or talin, and scaffold proteins (52, 53, 56, 57).

The Swiss-Model (58) predicts that EseG functions as a Rho-GEF. None of the EseG homologs, however, are reported to have similar host cell target proteins. The Rho-GEFs belong to a family of cytoplasmic proteins that activate the Ras-like family of Rho proteins by exchanging bound GDP for GTP (36). Rho GTPases control many aspects of cell behavior through actin/microtubule reorganization and regulation of multiple signal transduction pathways (32, 33, 38, 60–63).

RhoA GTPase functions as a molecular switch, regulating various cellular processes through its activation and inactivation. GEFs activate RhoA by promoting the exchange of GDP for GTP, while GAPs inactivate RhoA by enhancing intrinsic GTP hydrolysis. Additionally, RhoA can be kept in an inactive state by guanine nucleotide-dissociation inhibitors, which sequester prenylated GDP-bound Rho proteins and facilitate their translocation between the cytosol and membranes (38, 71).

Our data indicate that the *E. ictaluri* effector protein EseG can inactivate RhoA (Fig. 3) and interacts with RhoA (Fig. 4), though the exact mechanism remains unknown. Previous studies have shown that *Salmonella* effectors SseF and SseG inhibit autophagy by directly interacting with the Ras GTPase Rab1. This interaction disrupts Rab1 activation by interfering with the interaction with GEFs (72). Given the structural similarity of EseG to GEFs, it is possible that EseG may act similarly to *Salmonella* effectors by interacting with RhoA and preventing its activation by cellular GEFs. Further studies are needed to confirm this mechanism.

RhoA plays a critical role in regulating the dynamic organization of the actin cytoskeleton, influencing processes such as actin polymerization, actomyosin contractility, and stress fiber formation (59). These actin-based activities are fundamental for various cellular functions, including cell morphology, microtubule dynamics, polarity, adhesion, migration, motility, protrusion formation, exocytosis, endocytosis, cell cycle regulation, cytokinesis, neurite development, transcriptional control, and proliferation (73).

Our research indicates that the *E. ictaluri* T3SS effector, EseG, similar to its *E. piscicida* homolog EseG, destabilizes microtubule structures (Fig. 5). Effectors that disrupt microtubules include *E. piscicida* EseG, *Salmonella* effectors SseG and SseF, *E. coli* EspG, and *Shigella* effector VirA (8). Despite some progress in understanding the mechanisms of VirA and EspG, much remains unknown about how these other effectors cause microtubule disruption (8). Further research into the molecular mechanisms behind the microtubule disruption by effectors such as EseG will be essential for a comprehensive understanding of how pathogens manipulate the host cytoskeleton. In addition, tubulin reorganization can enhance vesicular transport within macrophages, supporting processes like phagosome formation and maturation, which are essential for killing internalized bacteria (74, 75).

EseG colocalized with the ECV and tubulin (Fig. 6) in infected HKDM. This colocalization suggests that these two proteins—EseG and tubulin—occupy the same spatial compartments at the same time, indicating a potential interaction or functional linkage within the infected cells. Interestingly, while *E. piscicida’s* EseG contains a tubulin-targeting domain, this domain is absent in *E. ictaluri’s* EseG. Despite this, we hypothesize that EseG may affect microtubule dynamics indirectly through its interaction with RhoA, which is known to regulate the cytoskeleton. Given that EseG interacts with and inactivates RhoA, and the protein complexes of EseG, RhoA, and tubulin localize within the same cellular compartments, it is plausible that this coordinated spatial relationship underpins microtubule disruption.

Rho family GTPases serve many cellular functions, including cell signaling, transcriptional regulation, and organization of the actin cytoskeleton. For example, signal transduction through Rho GTPases results in the reorganization of actin into stress fibers and the formation of focal adhesions (76, 77). Cell morphology and motility depend on the actin cytoskeleton regulated by Rho GTPases, including RhoA. It was demonstrated that the response of macrophages to RhoA inhibition varies depending on the macrophage subtype. The shape of macrophages is different depending on the subtype: naïve M0 macrophages are slightly elongated, inflammatory M1 macrophages are more round, and anti-inflammatory M2 macrophages are elongated. It was shown that RhoA pathway interference induced an elongated phenotype in M0 and M2, but not in M1 macrophages. It also inhibited the expression of M2-specific but not M1-specific molecular markers (78, 79).

Our data also indicate that EseG is involved in actin reorganization (Fig. 7) and changes in HKDM morphology after infection with *E. ictaluri* (Fig. 8A and B). Interestingly, HKDM infected with WT were more elongated, and HKDM infected with Δ*eseG* were more intermediate (Fig. 8B), suggesting that EseG can suppress inflammatory responses. It was also reported that nuclear actin plays a crucial role in chromatin remodeling complexes. By binding to RNA polymerase complexes and various transcription factors, it regulates gene expression, sequesters transcriptional activators and repressors, and controls processes such as transcriptional/nuclear reprogramming, cell differentiation, and developmental reprogramming (79). This can support changes in mRNA expression of the transcription factors SP2 and ATF3 (Fig. 9), and changes in pro-inflammatory interleukin expression (Fig. 10). Transcription factor SP2 typically promotes inflammatory gene expression. Reducing SP2 may lower the capacity of macrophages to produce the inflammatory mediators needed for bacterial clearance (80–82). ATF3 regulates responses to cellular stress, helping to manage inflammation. Reduced ATF3 could lead to a less responsive stress adaptation, possibly affecting macrophage survival under stress conditions related to infection (83, 84). Reduced SP2 and ATF3 may decrease inflammation further, dampening macrophage responses and limiting adaptation to stress within the infection environment. We found that *E. ictaluri* EseG modulates the host immune response by reducing pro-inflammatory interleukins IL-1, IL-6, IL-8, IL-12a, IL-16, IL-17, and IL-8 that support this hypothesis (Fig. 10). These cytokines play key roles in driving inflammation, recruiting immune cells, and supporting macrophage and neutrophil activity. Lower levels of IL-1 and IL-6 can reduce macrophage activation, potentially reducing bacterial clearance (85). IL-8 and IL-17 are crucial for attracting neutrophils, which play a primary role in bacterial clearance. Lower levels of these cytokines may result in a less effective innate immune response (86–88). IL-12α supports T-helper type 1 cells, differentiation and activation of cytotoxic T cells, and natural killer (NK) cells. Decreased IL-12α could reduce the adaptive immune response, especially the type of response needed to clear intracellular pathogens (89–91). As a result, reduced levels of these cytokines contribute to a generally low-inflammatory environment, which may be beneficial in limiting tissue damage but detrimental to efficient bacterial clearance, especially for intracellular pathogens. This could delay bacterial clearance and prolong infection. Interestingly, the only interleukin that was not repressed by the *E. ictaluri* WT strain was IL-15 (Fig. 10). Increased IL-15 expression could help sustain NK cell and CD8+ T cell activity, which might assist in targeting intracellular pathogens like *E. ictaluri* (92, 93). Any positive impact of increased IL-15 expression alone on the innate immune response to WT *E. ictaluri* infection, however, does not appear to seriously impact the disease process.

We also demonstrate that *E. ictaluri* effector EseG inhibits COX-2 and PGE2 production (Fig. 11). It has been demonstrated that PGE2 regulates macrophage production of pro-inflammatory and anti-inflamatory cytokines (94–96). These observations lead to the conclusion that PGE2 may participate in the inhibition of the host defense by deactivating macrophage responses against *E. ictaluri*. It was also demonstrated that IL-6 synthesis was stimulated by activation of COX-2 and production of endogenous PGE2 (97). Our data are consistent with this observation. Lower levels of PGE2, IL-1, IL-6, IL-8, and IL-17 in WT versus Δ*eseG* infection can lead to a less inflammatory environment, reducing neutrophil recruitment and macrophage activation (98–100). This makes it more difficult to mount a rapid response to bacterial invasion. This is further supported by the reduction in tissue numbers of *E. ictaluri* following infection with the Δ*eseG* strain compared to the WT, as well as by the resulting reduction in mortality (Fig. 12A and B).

In conclusion, this study demonstrates that *E. ictaluri* T3SS effector EseG has an important impact on *E. ictaluri* virulence using mechanisms that are not described for any EseG homologs. While some aspects support infection control, others may compromise the effectiveness of the overall immune response and make macrophages conducive to *E. ictaluri* intracellular replication. Immune and cytoskeletal profiles in macrophages during *E. ictaluri* infection suggest that EseG targets responses that prioritize cellular integrity, reduced inflammation, and enhanced phagocytosis. The reduced pro-inflammatory signaling by EseG may allow *E. ictaluri* to evade the immune response that relies on neutrophil and cytokine-driven elimination. *Edwardsiella ictaluri* may still be partially controlled by phagocytosis and IL-15-boosted cellular immunity, but clearance would likely be slower.

This work also has global significance for the aquaculture industry that provides seafood for a growing global population, and in which *E. ictaluri* is a leading cause of disease loss (1). Recently, Edwardsiellosis was reported in other cultured fish species, including *Oreochromis niloticus*, *Danio rerio, Brown bullhead*, *Ameiurus nebulosus,* and *Ayu* in Japan, as well as *Pangasius* catfish in Vietnam and China (101–103). Our data establish mechanisms of disease that suggest procedures for prevention through immune enhancement and vaccine development. This work will also help to identify functional domains in *E. ictaluri* T3SS effectors, which will provide knowledge to enable possible modulation of virulence and potentially predict virulence mechanisms for a variety of other animal and human pathogens. Finally, *E. ictaluri* T3SS effectors can become valuable tools for the general study of cell biology (104).

## MATERIALS AND METHODS

### *In silico* analysis

BLAST was used to align EseG in order to identify homologs in the sequence databases. ClustalX was used for multiple alignments of protein sequences, including EseG (GenBank accession number WP_263116232) and its homologs from *E. piscicida* (GenBank accession number AAX76916) formerly classified as *E. tarda*, *E. piscicida* (GenBank accession number WP_236893707), *Edwardsiella anguillarum* (GenBank accession number WP_117026488), *Salmonella enterica* subsp. salamae (GenBank accession number HAU3358950), and *S. enterica* subsp. enterica (GenBank accession number EBP3474089) in order to identify conserved domains. Function-specific conserved domains were identified based on published work (8).

### Bacterial strains, plasmids, and media

Strains and plasmids used in this study are listed in Table 1. *Edwardsiella ictaluri* strains were grown at 28°C on either trypticase soy agar plates supplemented with 5% sheep blood (BA; Remel Products, Lenexa, KS) or porcine brain heart infusion (pBHI) agar (BD Difco, Lawrence, KS). Broth cultures of *E. ictaluri* were grown in either porcine BHI broth (pBHIB) or *E. ictaluri* low-phosphate minimal medium (105, 106). *Escherichia coli* strains were cultured using Luria-Bertani (LB) broth or agar at 37°C (BD Difco). All strains grown in broth were aerated on a Max Q4450 incubated shaker (Thermo Scientific, Marietta, OH, USA). Antibiotics were added where appropriate at 200 µg/mL for ampicillin (Amp) and 20 µg/mL for colistin (Sigma).

**TABLE 1:** Bacterial strains and plasmids used in this study

Strain or plasmid	Relevant characteristic(s)	Reference or source
Strains		
*E. coli* S17 λ-pir	(F−) RP4-2-Tc::Mu *aphA*::Tn7 *recA* λ-pir	(107), ATCC
*E. ictaluri* 93-146	Wild-type *E. ictaluri* isolated from a moribund channel catfish from a natural outbreak at a commercial facility in 1993	LSU aquatic diagnostic laboratory
*E. ictaluri* 65ST	93-146 esaU::Tn5Km2	(5)
*WT/eseG*::*cyaA*	Wild-type *E. ictaluri* carrying BBR1, *eseG::cyaA* Amp^r^	This work
*WT/escBeseG::cyaA*	Wild-type *E. ictaluri* carrying BBR1, *escBeseG::cyaA*	(7)
*∆escB/ eseG::cyaA*	*escB* mutant carrying BBR1, *eseG::cyaA*	This work
*∆escB/ escBeseG::cyaA*	*escB* mutant carrying BBR1, *eseG::cyaA*	This work
*∆eseG*	*E. ictaluri* 93-146 Δ(*eseG* 1-773)	(7)
*∆escB*	*E. ictaluri* 93-146 Δ(*escB* 1-499)	This work
*∆escG/ eseG::3flag*	*eseG* mutant complemented with *eseG::3flag*	This work
*∆escG/ eseG*	*eseG* mutant complemented with *eseG*	(7)
Plasmids		
pMJH20	Plasmid containing CyaA adenylate cyclase	(108)
pRE107	Suicide plasmid for allelic exchange	(109)
pBBR1MCS-4	Broad-host-range expression vector	(110)
pBBR1-*eseG::cyaA*	pBBR1MCS4 carrying *eseG::cyaA*	This work
pBBR1-*escBeseG::cyaA*	pBBR1MCS4 carrying *escBeseG::cyaA*	(7)
pRE107-*ΔescB*	pRR107 with *escB* deletion	This work
pRE107-*eseG::3flag*	pRR107 with *eseG::3flag* complementation	This work
pBBR1-*eseG*	Complementation plasmid	(7)

### Specific pathogen-free (SPF) channel catfish

Channel catfish egg masses obtained from production facilities with no history of *E. ictaluri* outbreaks were disinfected with 100 ppm free iodine and were hatched and reared in closed recirculating aquaculture systems in the SPF aquatic laboratory at the LSU School of Veterinary Medicine. Catfish used for harvesting macrophages were reared in the SPF lab and weighed between 500 and 750 g each.

### Preparation and infection of head kidney-derived macrophages

The isolation of HKDMs was performed as previously described (4). Head kidneys were obtained from SPF channel catfish, and head kidney cells were seeded in six-well plates for RNA isolation and into four or eight chambered microscopic slides for confocal microscopy. For *E. ictaluri* infection, bacterial strains were opsonized for 30 min in normal autologous serum and added to duplicate wells of HKDM for microscopic assays and in quadruplicate wells of HKDM for RNA analysis at a multiplicity of infection (MOI) of 10 bacteria to 1 HKDM. Uninfected cells were used as a negative control. After infection, plates were centrifuged at 500 × *g* for 5 min to synchronize contact of the bacteria with the adhered cell layer and were allowed to incubate for 30 min, after which a killing dose of 100 µg/mL gentamicin was added and cultures were incubated at 28°C for 1 h to remove any remaining extracellular bacteria. Finally, wells were washed once with channel catfish RPMI (ctRPMI) (4), and channel catfish macrophage media (4) containing a 1 µg/mL bacteriostatic dose of gentamicin was added to control any extracellular growth of bacteria released from the cells during the experiment.

### DNA manipulations

DNA manipulations were performed by standard methods. All enzymes used in plasmid construction were obtained from New England Biolabs (Beverly, MA). Total DNA was purified from cultures using the High Pure PCR template preparation kit (Roche, Mannheim, Germany), and Phusion high-fidelity polymerase (Thermo Scientific, Waltham, MA) was used for PCR amplifications. DNA restriction fragment isolation and PCR product purification were done using the QIAquick gel extraction kit (Qiagen, Valencia, CA). All procedures were performed according to the manufacturer’s instructions. Oligonucleotide primers used in this study were designed based on coding sequences from the *Edwardsiella ictaluri* 93-146 reference genome (RefSeq NC_012779.1) or the *Ictalurus punctatus* genome (GenBank accession no. PRJNA281269). Specificity was confirmed using NCBI Primer-BLAST against the appropriate genome to ensure target specificity and preclude off-target amplification. Oligonucleotide primers were purchased from Integrated DNA Technologies (Coralville, IA). All constructs were confirmed by PCR and DNA sequencing.

### Construction of *eseG*::3Xflag, *escBeseG*::cyaA fusion, and *escB* mutant

The strategy for constructing the effector-CyaA fusions was described previously (7); specific primers used in this study are listed in Table 2, as are specific primers for the other constructs. The *eseG* construct lacking *escB* was amplified from *E. ictaluri* genomic DNA with a gene-specific forward primer, eseG P1, that annealed 264 bp upstream from the translational start codon of *escB* so that the native promoter and ribosomal binding site (RBS) of escB were included along with a linker containing an XbaI restriction enzyme site to facilitate cloning. Primer P1 was paired with the reverse primer, P2, which included a linker that contained 27 bp to amplify the *eseG* gene (underlined). The 27 bp link of P2 contains 16 bp of intergenic *escB eseG* sequence that contains the RBS sequence for *eseG* and 11 bp of the *eseG* gene starting from the start codon.

**TABLE 2:** Primers used for cloning^*a*^

Primers	Forward (5´−3´)	Enzyme site	Reference or source
	**Primers used to construct *eseG::cyaA* fusion**		
eseG P1	GTACGCTCGAG**TCTAGA**TCGTCTAGAATCGGGCGCTGGATAAGATGCGACGACGCCTGAC	XbaI	(7)
P2	CCAGTGATCATCATGGGCTCCTTAACGTAAAGAGTCTGATATGTTATTACATTATCAGG		This work
P3	CCTGATAATGTAATAACATATCAGACTCTTTACGTTAAGGAGCCCATGATGATCACTGG		This work
eseG P2	GCGTAACCAGCCTGATGCGATTGCTGAAAGAAGCATGCGGCAAAGCTGTGGCGTCGTGTC		(7)
eseG P3	GACACGACGCCACAGCTTTGCCGCATGCTTCTTTCAGCAATCGCATCAGGCTGGTTACGC		(7)
P4	GAGCGTACC**TCTAG*A****AAAAATGGGGGATAACACCCCCATT*ATTGGCGTTCCACTGCGCCCA GCGACGGCCGCCGCCGCAATCCGGGTG	XbaI	(7)
	**Primers used to construct *escB* mutant**		
eseG P1m	GAATCGTGTACAGG**GTCGAC**CACTTCCTGGAGAGTGAGTTTCTCATCGGGCAG	Sall	This work
eseG P4	GATGCC**TCTAGA**TGGCAAAGCTGTGGCGTCGTGTCAGT	XbaI	(7)
P2	CCAGTGATCATCATGGGCTCCTTAACGTAAAGAGTCTGATATGTTATTACATTATCAGG		This work
P3	CCTGATAATGTAATAACATATCAGACTCTTTACGTTAAGGAGCCCATGATGATCACTGG		This work
	**Primers used to construct *eseG::3flag***		
eseG P1	GTACGCTCGAG**TCTAGA**TCGTCTAGAATCGGGCGCTGGATAAGATGCGACGACGCCTGAC	Xba I	(7)
eseG::3flag P2	ttaCTTGTCGTCATCGTCTTTGTAGTCCTTGTCGTCATCGTCTTTGTAGTCCTTGTCGTCATC GTCTTTGTAGTCggcaaagctgtggcgtcgtgtcagtg		This work
eseG::3flag P3	GACTACAAAGACGATGACGACAAGGACTACAAAGACGATGACGACAAGGACTACAAAGAC GATGACGACAAGtaaTGAGCCAGTCTACCGCCTCATGGACA		This work
eseG P4	CACGATGCC**TCTAGA**TACTGACGGTTTCACGGTTTTGTTCCTGGTTAAGA	XbaI	(7)

^*a*^ Bold text represents restriction sites.

The *eseG* gene was amplified with primers P3 and eseGP2. Primer P3 is the reverse complement of P2. Primer eseGP2 included a linker that contained 26 bp of the adenylate cyclase (AC) domain of CyaA (underlined). The 1,197 bp AC domain of CyaA from bp +4 to +1,227 was amplified from the plasmid pMJH20 (108) with primer eseG P3, which included 22 bp of *eseG*, and primer P4, which included an in-frame stop codon and the rho-independent transcriptional terminator (italic) from the *Bacillus subtilis yqfT* gene (111), and another XbaI site. To produce the fusion constructs, the promoter, effector, and CyaA amplicons were mixed and amplified using primers eseG P1 and P4, and the construct was cloned into the plasmid pBBR1MCS-4 (110). The resulting plasmids were transformed into *E. coli* S17-1 λ-pir (107) and transferred to *E. ictaluri* by conjugation (112).

The ∆*escB* mutants were constructed in a similar manner except that primers eseGP1m and P2 amplified from 486 bp upstream of the *escB* start codon through the RBS of *escB*, and primers P3 and eseG P4 amplified from 16 bp of the intergenic sequence between *escB* and *eseG* genes containing the *eseG* RBS sequence to the end of the *eseG* gene. Primers eseGP1m and eseG P4 contained restriction endonuclease sites to facilitate final cloning of the gene-deleted fragment. Primer P2 included overlapping sequence of the right arm, and P3 contained overlapping sequence of the left arm so that when the PCR products were mixed, they annealed to each other; amplification with the eseGP1m and eseGP4 primers resulted in a fragment with the *escB* deletion and flanking sequence on either side of *escB* deletion to mediate integration of the plasmid into the chromosome. Primers used for the construction of the *escB* mutant are listed in Table 2. The deletion constructs were cloned into the suicide plasmid pRE107 (109), transformed into *E. coli* S17 λ-*pir*, transferred to *E. ictaluri* 93-146 by conjugation, and grown in pBHI-Amp to select for plasmid integration into the chromosome. Colonies positive for Amp^r^ were then grown in pBHI with 7.5% sucrose to select for a second crossover and excision of the plasmid, which resulted in a mixture of wild-type and deletion mutants. Deletion mutants were identified by PCR using effector-specific primers and confirmed by DNA sequencing. The final mutant construct consisted of a complete gene deletion of *escB*.

The *eseG::3flag* strain was also constructed by PCR. Primers eseG P1 and eseG::3flag P2 amplified 784 bp of the EseG flanking sequence to the end of the *eseG* gene. Primers eseG::3flag P2 and eseG::3flag P3 contain three flag sequences and a stop codon. Primers eseG::3flag P3 and eseG P4 amplified from the carboxy terminus of *eseG* to bp 1,025 of the flanking sequence. Primers eseG P1 and eseG P4 contained selected restriction endonucleases to facilitate final cloning. Primer eseG::3flag P2 included overlapping sequence of the right arm, and eseG::3flag P3 contained overlapping sequence of the left arm so that when the PCR products were mixed, they annealed to each other; amplification with the eseG P1 and eseG P4 primers resulted in a fragment with the required construct and with flanking sequence on either side of the effector to be inserted to mediate integration of the plasmid into the chromosome. Primers used for the construction are listed in Table 2. The resulting constructs were cloned into the suicide plasmid pRE107 (109), transformed into *E. coli* S17 λ-*pir*, transferred to *E. ictaluri* ∆*eseG* by conjugation, and grown in pBHI-Amp to select for plasmid integration into the chromosome. Colonies positive for Amp^r^ were then grown in pBHI with 7.5% sucrose to select for a second crossover and excision of the plasmid, which resulted in a mixture of *eseG::3flag* complement and *E. ictaluri* ∆*eseG*. The *eseG::3flag* strain was identified by PCR using effector-specific primers and confirmed by DNA sequencing.

### Translocation assay

To investigate the role of EscB in the translocation of EseG into the cytosol of HKDM cells, several mutants and constructs were generated. EseG was fused to the N-terminal AC domain of the *Bordetella pertussis* CyaA toxin, a well-established reporter for studying T3SS effector translocation (108, 113–115). This assay relies on measuring cAMP produced by the interaction of the translocated AC domain with calmodulin, which is present in the HKDM cytoplasm but not in the ECV. The EseG::CyaA fusion, with or without its chaperone, was cloned into the plasmid pBBR1MCS-4 (110) and transformed into *E. ictaluri* WT or Δ*escB* strains.

Translocation of EseG-CyaA fusions from *E. ictaluri* in the ECV to the cytoplasm was evaluated using HKDM cells by measuring cAMP production. Briefly, duplicate wells of HKDM were infected at an MOI of 10 bacteria to 1 HKDM. Following infection, any remaining extracellular bacteria were killed using 100 µg/mL of gentamicin. At 7 h PI, cells were lysed with 0.1 M HCl containing 0.1% Triton X-100. Levels of cAMP produced by the interaction of the AC domain of the CyaA toxin with calmodulin in the host cell cytoplasm were measured in picomoles of cAMP per milliliter by using the cAMP complete enzyme-linked immunosorbent assay (ELISA) kit from Enzo Life Sciences (Plymouth Meeting, PA). Production of cAMP was normalized by determining the protein concentration in each sample using the Bio-Rad protein assay and calculating the number of picomoles of cAMP per milligram of protein.

### RT-qPCR

Quantitative RT-qPCR was performed using the total RNA extracted from the HKDM infected with *E. ictaluri* strains for 1, 3, 5, and 7 h PI, using RNAzol RT Isolation Reagent (Molecular Research Center, Cincinnati, OH, USA) in combination with the Direct-zol RNA MiniPrep (ZYMO Research, USA), which was used for DNase treatment and washing steps only, following manufacturer protocols. The RNA samples were resuspended in molecular-grade water (Ambion, TX, USA), aliquoted, and stored at −80°C until use. The RNA concentration and purity were determined using Nanodrop (BioTek Synergy LX Multi-Mode Reader, Daytona Beach, FL, USA) with software Gen5 version 3.11.

The qPCR assays were carried out by qPCRBIO SyGreen 1-Step Go Lo-ROX kit (PCR Biosystems, Wayne, PA, USA). One-step qPCR was performed using 10 ng of each RNA sample, 0.5 µM of each gene-specific primer (Table 3) at 54°C for 10 min, 95°C for 2 min, and 40 cycles of 95°C for 5 s and 61°C for 30 s in a LightCycler 96 System (Roche Applied Science, Indianapolis, IN, USA). Oligonucleotide primers were purchased from Integrated DNA Technologies (Coralville, IA, USA). The gene CanX was used as a reference gene (14, 16).

**TABLE 3:** Primers used for RT-qPCR

Primers	Forward (5´−3´)	Reverse (5´−3´)	References
CanX	GCTGTTAAACCGGAGGACTG	GCAGGTCCTCGAAGTAGTCAG	(14)
IL17C	CTC CTT TTC CTC ACG GAT GCT	CCG TCC TCC GCT GTA ATC TC	This study
ATF3	AGA GTA AAC GCC TGT CCA GC	TCC CGT CGT CTT TTC CTA CG	This study
IL-1	GTC CTG ACT GTC GAG TGC TG	CGG CAG TTG TCC TTC GTA CA	This study
SP2	GGG ATC GAG TCA GGG TGT TC	CGC TCG TCT GGA TCG ACT AC	This study
IL-12a	AGG ACT GAG GAA CCA TGC AC	TTT TGG GGT CTG TTG TGG TGT	This study
IL-16	CTG TAT GGA GCG AAG CCA CA	GTA AGC ACC CTC GCT ACA GG	(14)
IL-10	CTC CTC CCC CTG AGG ATT CA	CGG ATC ACG GCG TAT GAA GA	(16)
IL15	CGG CGA TTT GTT CGA TGC AG	CTC CTG GTT CAA GGG TCA C	(14)
IL16	CAT CAG TCG ACA CCC TGA C	CTG TGG CTT CGC TCC ATA C	(14)
IL-8aII	GAT TGC TCA ACC TTT TCG CAT TAC	CAT GGC CTG TGA TTT AGC TGT G	This study
COX2	CAG GTC GAG ATG CAC TAC C	GTA GTA GCC GCT CAG GTG	(14)

### Immunofluorescence and confocal assay

Channel catfish HKDMs were obtained and infected as described above. Following 5 h post-infection, the infected macrophages were fixed with 4% paraformaldehyde in phosphate-buffered saline (PBS) for 20 min and permeabilized with 0.1% Triton X-100 in PBS for 5 min. After washing in PBS, the cells were blocked with 2.5% bovine serum albumin (Sigma-Aldrich) and 2% goat serum (Thermo Fisher Scientific) at room temperature for 1 h.

After additional washing, the cells were incubated with primary antibodies overnight at 4°C. The slides were then washed with PBS and incubated for 1 h with Alexa Fluor-conjugated secondary antibodies (Abcam) specific to the primary antibody. Finally, a mounting medium containing DAPI was applied for nuclear staining.

For microtubule destabilization, rabbit anti-α/β-tubulin antibodies (Cell Signaling Technology) were used, while detection of *E. ictaluri* used the monoclonal antibody Ed9 (4, 5, 116). Similarly, for colocalization analysis of EseG with microtubules, anti-α/β-tubulin antibodies (Cell Signaling Technology) were used for tubulin detection, the monoclonal antibody Ed9 (4, 5, 116) was used for *E. ictaluri* detection, and chicken polyclonal anti-DDDDK tag antibody (ab1170, Abcam) was used for EseG detection.

To observe actin reorganization, *E. ictaluri* was detected in infected HKDM cells with the Ed9 monoclonal antibody (4, 5, 116) overnight at 4°C, followed by staining with the secondary anti-mouse antibody, and phalloidin (Phalloidin-Fluor 594 Reagent; ab176757) was used to detect F-actin.

Images were captured using an Olympus FluoView FV10i confocal microscope. Representative areas of each slide were imaged, and more than 100 infected cells per condition were analyzed across three to four independent experiments. Microtubule destabilization was quantified by counting over 100 infected cells per condition to assess the disappearance of tubulin structures (28).

Colocalization was determined visually (117, 118). For colocalization analysis, regions of interest (ROI) were selected around infected cells, and signal overlap between EseG::FLAG and tubulin and between EseG::Flag and *E. ictaluri* was quantified and analyzed using the FV1000-ASW software.

Pearson’s correlation coefficient (*R*) was used to assess the correlation between fluorescence intensities of the two channels across all pixels within each ROI. Manders’ overlap coefficient (*M*) was used to quantify the proportion of each fluorophore’s signal overlapping with the other, regardless of signal intensity correlation. For control comparison, WT untagged samples were used as negative control (117).

### Proximity ligation assay

Interactions between EseG and RhoA were detected by using the Duolink PLA kit according to the manufacturer’s instructions (Sigma-Aldrich). In brief, HKDMs were seeded in eight-well chamber slides (ThermoFisher Scientific) and infected with WT and ΔEseG–ΔeseG::Flag strains. After 5 h PI, cells were fixed, permeabilized, and blocked as described above.

The mouse monoclonal antibody (MAb) anti-FLAG M2 (Sigma-Aldrich) and rabbit polyclonal anti-RhoA (Abcam) were used as primary antibodies, and anti-rabbit and anti-goat PLA probes were used as secondary antibodies. Sites of protein-protein interaction were detected as red fluorescent spots on an Olympus Fluoview FV10i confocal microscope using Texas Red filters for PLA fluorophore detection and a DAPI filter to visualize the nuclei.

### ELISA for RhoA activation

Channel catfish HKDMs were seeded into wells of 24-well plates containing complete ctRPMI medium, and the infection was performed in serum-free medium with *E. ictaluri* WT or Δ*eseG* strains as described above. Serum-free medium ctRPMI was used for this experiment to avoid RhoA activation by serum. After 5 h PI, cells were lysed, and RhoA activity was measured using the RhoA G-LISA Activation Assay Kit (Cytoskeleton, Inc.) according to the manufacturer’s instructions. The absorbance was recorded with a Spectra Max M2 microplate reader (Molecular Devices, Sunnyvale, CA, USA).

### *In vivo* virulence evaluation of ΔeseG *E. ictaluri*

#### *In vivo* invasion and tissue persistence

To evaluate the ability of *E. ictaluri* strains to invade and replicate in the channel catfish head kidney, 20 L tanks receiving 0.5 L min^−1^ of dechlorinated Baton Rouge city water were stocked with 25 fish (10–12 cm, 4–6 months old) each, and quadruplicate tanks of each treatment were challenged by immersion for 30 min with either pBHIB (control), WT, Δ*eseG*, or 65ST *E. ictaluri* strains at final concentrations of 2.5 × 10^8^ CFU/mL for WT, 3.0 × 10^8^ CFU/mL for Δ*eseG*, and 3.0 × 10^8^ CFU/mL for 65ST. A single fish from each replicate tank was removed and euthanized in 1 g/L tricaine methanesulfonate (MS-222) at 8 h PI and then daily for 5 days. To evaluate the number of CFU per gram of tissue, approximately 100 mg of head kidney tissue was removed, weighed, homogenized in 100 µL of PBS, spread on blood agar (BA) plates, and incubated at 28°C for 48 h. Colonies were counted, and the number of CFU recovered per gram of tissue was calculated.

#### Channel catfish infection challenges

Twenty-liter tanks as described above were stocked with 25 SPF fish/tank and acclimated for 3 weeks. Water flow to the tanks was interrupted, but aeration was maintained, and fish in quadruplicate tanks were challenged by immersion with each *E. ictaluri* strain at a final concentration of 1.3 × 10^8^ CFU/mL for WT, 8.4 × 10^7^ CFU/mL for Δ*eseG,* and 9.8 × 10^7^ CFU/mL for Δ*eseG*/*eseG*. Fish exposed only to pBHIB served as negative controls. After a 1 h exposure, water flow was restored, and the challenge dose was slowly replaced with fresh water. Moribund and dead fish were collected daily, and the presence of *E. ictaluri* was confirmed by plating liver tissue from each mortality onto BA plates. Sampling continued until 3 days passed without a death in any treatment.

### Statistical analysis

All data analyses were performed using the GraphPad Prism 5.02 software (GraphPad Software, Inc., La Jolla, CA). Most analyses were conducted using one-way analysis of variance (ANOVA) followed by the Bonferroni *post hoc* test for pairwise comparisons. Persistence of the WT and mutant strains in the tissue following immersion exposure was analyzed using two-way ANOVA followed by Bonferroni’s *post hoc* test to determine significant differences between WT and mutant strains of *E. ictaluri*. Percent mortality data were analyzed following an arcsine transformation of the percentage values. The *t*-test was used when comparing only two groups.
